# Alpha-1 antitrypsin deficiency in the elderly: a case report

**DOI:** 10.1186/s13256-021-02847-w

**Published:** 2021-05-10

**Authors:** Anna Annunziata, Maurizia Lanza, Antonietta Coppola, Giuseppe Fiorentino

**Affiliations:** UOC Pathophysiology and Respiratory Rehabilitation, Intensive Care Department, Monaldi Hospital, Naples, Italy

**Keywords:** Alpha-1 antitrypsin, Replacement therapy, Lung disease, Asthma

## Abstract

**Background:**

Generally, alpha-1 antitrypsin deficiency (AATD) is suspected in young patients with pulmonary emphysema or chronic obstructive pulmonary disease (COPD). Patients often suffer from diagnostic gaps and are misdiagnosed with chronic obstructive pulmonary disease (COPD), asthma, and airway hyperresponsiveness (AHR), as AATD may present with nonspecific respiratory symptoms. It is never too late to suspect AATD, especially in a patient with an unusual medical history. In recent years, evidence is beginning to emerge that there may be value in identifying and treating patients who do not already have deterioration of functional parameters.

**Case presentation:**

We describe a case of a 69-year-old Caucasian female patient, late diagnosis of AATD, with both severe bronchial hyperreactivity and numerous exacerbations due to the peculiar clinical history and the presence of a rare mutation; although not presenting forced expiratory volume in 1 second (FEV_1_) between 30 and 65%, the patient was treated with alpha-1 antitrypsin (AAT) augmentation therapy and achieved clinical and functional improvement.

**Conclusion:**

AATD should always be suspected. The Alpha-1 Foundation recommendations for the diagnosis and management of AATD in adult patients indicate that treatment should be provided for patients with FEV_1_ between 30 and 65%. It may be useful to evaluate and treat patients based on clinical symptoms, even outside the established parameters, in particular cases.

## Background

Alpha-1 antitrypsin deficiency (AATD) causes different respiratory manifestations, including pulmonary emphysema, chronic obstructive pulmonary disease (COPD), asthma and bronchiectasis. AATD is a rare lung disease, with a progressive clinical course, and early diagnosis enables patients to make lifestyle changes and initiate therapy to decelerate or prevent further loss of lung tissue. However, AATD goes largely unrecognized; evidence from screening programs in the United States shows that less than 10% of individuals with AATD are clinically diagnosed with the disorder [1]. Patients often suffer from diagnostic gaps and are misdiagnosed with COPD, asthma and airway hyperresponsiveness (AHR), as AATD may present with nonspecific respiratory symptoms. Some authors have suggested that AHR, wheezing and dyspnea may be susceptibility phenotypes for the development of respiratory disease, especially in the context of cigarette smoking. In their group of patients, 25% had an increase of at least 10% in forced expiratory volume in 1 second (FEV_1_), and this reversibility has been associated with lower lung functionality [2]. Emphysema or COPD may be initially and incorrectly diagnosed as asthma in adults with AATD; a diagnosis of childhood asthma would be unlikely due to emphysema or COPD. In Sweden, individuals with AATD have been identified as part of neonatal screening; 15% were diagnosed with asthma by age 22, and 29% had self-reported recurrent wheezing episodes. The study noted that a medical diagnosis of asthma before age 16 was linked to reduced FEV_1_ and severe COPD in adulthood, which could indicate that asthma or asthma-like symptoms may result in a subset of particularly susceptible AATD patients [2]. Therapeutic diagnostic delay can lead to a complex clinical course.

## Case presentation

We describe the case of a 69-year-old Caucasian female patient, housewife and mother of three daughters, in apparent good health, who had never smoked. The patient reported six episodes per year of exacerbation of respiratory failure documented over the previous 10 years. Respiratory symptoms were resolved with steroids and antibiotics. The patient had been hospitalized twice in the last 3 years for acute respiratory failure. She was discharged with a diagnosis of "acute respiratory failure exacerbated by COPD.”

Due to persistence of respiratory symptoms characterized by dyspnea and a dry cough, the patient visited the pulmonary clinic of our department, and a functional assessment was carried out. At a young age, she reported occasional wheezing, sporadic episodes of respiratory exacerbation, especially during the three pregnancies, and intense dyspnea and asthma-like symptoms. She had undergone spirometry several years earlier, for which no written documentation was generated. The patient was being treated with an inhaled corticosteroid and long-acting β2-agonist twice daily, long-acting muscarinic antagonist twice daily, theophylline 200 mg orally twice a day, and montelukast 10 mg per day orally.

**Fig. 1:**
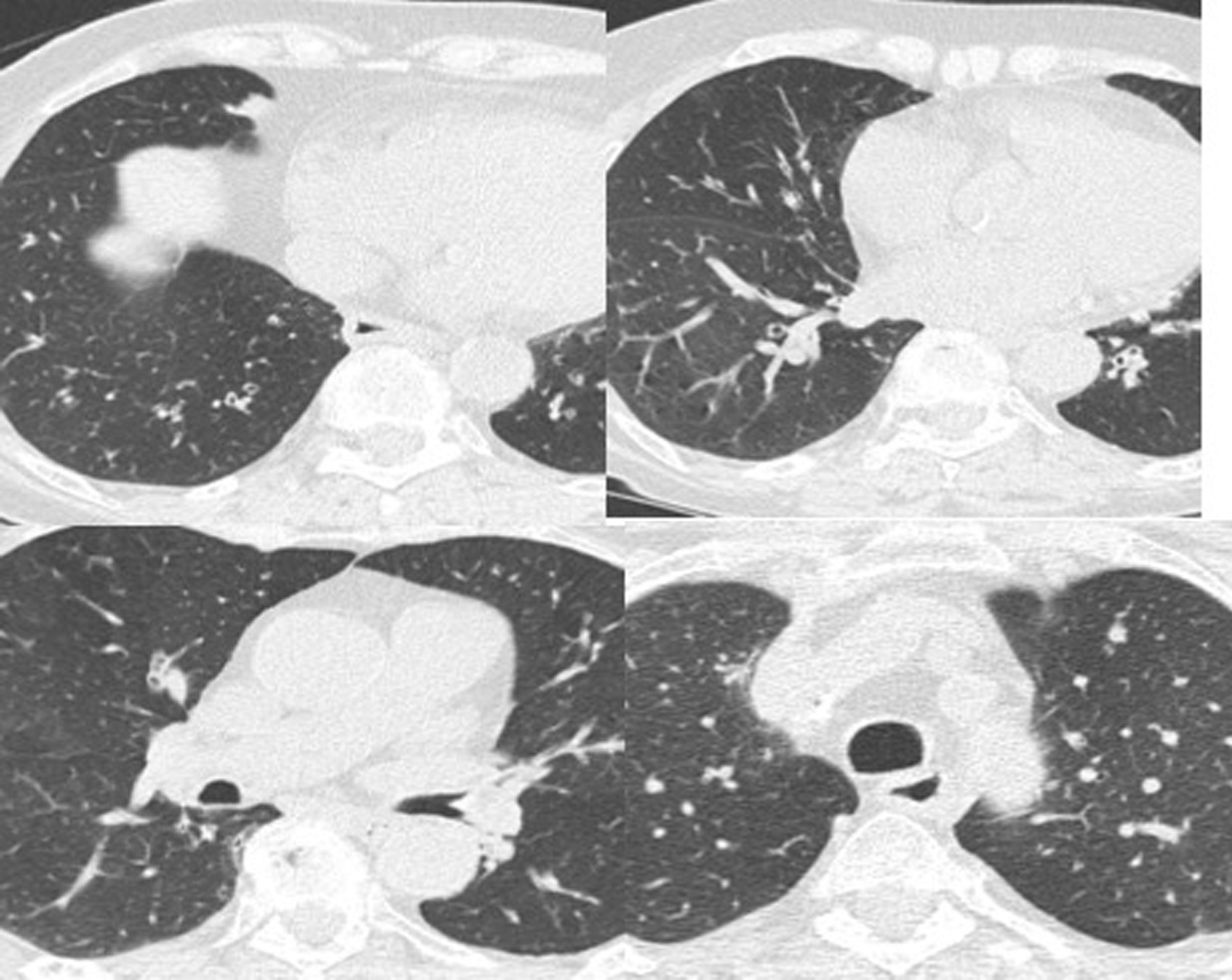
Chest computed tomography scan showed the presence of small areas of air trapping and diffuse bronchiectasis

The patient complained of worsening dyspnea with wheezing. Spirometry performed at the first visit showed forced vital capacity (FVC) of 2.17 (120%), FEV_1_ 1.64 (113%) and diffusing capacity for carbon monoxide (DLCO) of 4.11 mmol/(minute KPa), 80%. Spirometry performed at the second visit showed FVC 1.95 (100%), FEV_1_ 1.44 (100%), Tiffeneau index of 75% and DLCO 4.11 mmol/(minute KPa), 80%. The six-minute walk test was normal. She was subjected to a methacholine challenge test, which was positive for severe hyperreactivity. Allergic skin tests for inhalants and foods were negative. A paper radioimmunosorbent test (PRIST) showed a level of 39 U/mL. The blood tests were normal except for serum alpha-1 antitrypsin (AAT) of 67 mg/L (normal value is 0.90–2.00 g/L). Chest computed tomography scan revealed the presence of small areas of air trapping and diffuse bronchiectasis (Fig. 1). Sputum cultures performed to exclude colonization of potentially pathogenic microorganisms that could be the cause of exacerbations were negative.

Genetic screening was performed for the AATD inheritance and showed compound heterozygosity for deficient alleles pis and Pi_Lowell_.

After informed consent was obtained from the patient, we started intravenous augmentation therapy with human AAT at a dosage of 60 mg/kg every 7 days. At the request of the patient and her family, and after having monitored serum AATD, therapy was performed every 14 days. At 20 months of treatment, the patient showed no exacerbation, dyspnea exertional resolution and stable improvement in respiratory parameters.

After subsequent revaluation diagnostics, this patient, with a rare double heterozygous mutation, was treated with augmentation therapy, obtaining clinical and functional improvement without exacerbation over 2 years of treatment.

## Discussion

A review by Eden [3] examined the contention that individuals with severe AATD were particularly susceptible to AHR, which was associated with the reactivity of bronchodilators, asthma and allergies. This review summarizes evidence to suggest that the development of AHR related to asthma and reversible bronchospasm in AATD is a risk factor for progression to COPD. Different genetic factors are involved in the development of asthma, such as atopy, and increase the predisposition to the disease [3]. The different phenotypes of asthma depend on the interaction between heredity and the polygenic environmental complex [4]. Atopic asthma originates from a genetic predisposition, characterized by allergic manifestations, response to skin tests, eczema and rhinitis, and the response to triggering actors can cause attacks of wheezing and shortness of breath. Gastroesophageal reflux can also trigger AHR in susceptible individuals. AATD as a predisposing factor may contribute to the pathogenesis of asthma due to the lack of anti-neutrophil elastase of the airways; this results in a condition of chronic inflammation which can stimulate an acute reaction in response to various agents [5]. Asthma exacerbation can be caused by upper respiratory tract infection, obesity or exposure to allergens, including in the workplace [6, 7]. With airway inflammation of neutrophils in asthma and chronic bronchitis, both total and active proinflammatory elastase increases in the airways. There is a compensatory development in airway AAT, but an excess of elastase indicates protease/anti-protease imbalance [8]. Studies have revealed that the presence of asthma with frequent exacerbations is a risk factor for an accelerated decline in FEV_1_ [9, 10].

For over 10 years, the patient had respiratory symptoms variously diagnosed as episodes of acute bronchitis or chronic bronchitis. There were no professional or lifestyle risk factors, and the patient had never smoked; thus AATD was never suspected. However, despite maximizing drug treatment, the clinical conditions worsened, with an increase in the frequency of episodes of bronchial exacerbation. In the most recent few months, exertional dyspnea had arisen, but the basal functional evaluation and allergology tests carried out on the occasion of different re-evaluations determined nothing pathological. At the time of our first evaluation, the finding of lower than normal serum AAT level necessitated a genetic study documenting the heterozygous presence of a pis and Pi_Lowell_ mutation. The evolution of lung disease in people with AATD would appear to be aggravated by the presence of gene interactions that are not yet clearly understood. Presumably, atopy, modifier genes and extrinsic influences, environmental factors and cigarette smoking work together to increase airway hyperreactivity and asthma, contribute to the decline of lung function in AATD, and play a role in exacerbation [11].

It is interesting to observe that in our case, the patient at the second evaluation showed a significant reduction in respiratory function compared to her previous parameters, while remaining in the normal range of respiratory function. During the months of augmentation therapy, our patient did not show any bronchial exacerbation; the dyspnea index was reduced as assessed by the six-minute walk test and modified Medical Research Council (mMRC) scale (Table 1). The role of the distinct mutations of the gene for AAT and their combination is still an ongoing study. Different clinical pictures of different severity are described in the literature in patients with the same mutation or with rare mutations in combination. Serum AAT levels should be measured at least once in all patients with asthma and COPD, as suggested by the World Health Organization (WHO), as a large number of patients receive the diagnosis too late for effective treatment measures [12]. The latency time for diagnosis of congenital AATD is greater than 10 years. According to the Italian registry data, the diagnostic delay is about 8–9 years, which is greater than that for the United States and Germany (about 6 years) [13].

**Table 1.** Respiratory parameters measured during visit 1 and visit 2

	FEV_1_	FVC	po_2_	mMRC scale	6MWT
Visit 1	1.64 (113%)	2.17 (120%)	69	2	340
Visit 2	1.44 (100%)	1.95 (100%)	60	4	270

FEV1 forced expiratory volume in 1 second, FVC forced vital capacity, pO2 partial pressure of oxygen, mMRC modified Medical Research Council, 6MWT six-minute walk test

## Conclusion

These data strongly support a recommendation to always suspect AATD in the presence of respiratory symptoms at any age, and above all to promote greater awareness of this genetic condition, which is still under-recognized more than 50 years after its discovery. The exclusion of a genetic component linked to AAT is indicated in all patients with respiratory symptoms, asthma or COPD, and it is important to evaluate each case for the possible indication and potential advantages of AAT augmentation therapy.

The latest Alpha-1 Foundation recommendations for the diagnosis and management of AATD in adult patients supports replacement therapy in individuals with FEV_1_ of 30–65% [14], but emerging data showing that it can be useful for diagnosis and treatment of patients outside of previously established parameters [15]. Data on these cases need to be collected to better understand the role of augmentation therapy. Large-scale screening in the general population, infants and blood donors, or in targeted populations such as COPD and asthma patients, which can lead to the early identification of patients in need of treatment, would be helpful. Although AAT augmentation therapy is the only treatment available that addresses the underlying cause of AATD, symptomatic therapy and lifestyle changes may be of benefit to all patients.

## Data Availability

All data are available c/o UOC Pathophysiology and Respiratory Rehabilitation, Intensive Care Department, Monaldi Hospital, Naples, Italy.
